# An antagonist of retinoic acid receptors more effectively inhibits growth of human prostate cancer cells than normal prostate epithelium

**DOI:** 10.1038/sj.bjc.6602024

**Published:** 2004-07-13

**Authors:** R G Keedwell, Y Zhao, L A Hammond, K Wen, S Qin, L I Atangan, D-L Shurland, D M A Wallace, R Bird, A Reitmair, R A S Chandraratna, G Brown

**Affiliations:** 1 Divisions of Immunity and Infection, University of Birmingham Medical School, Edgbaston, Birmingham B15 2TT, UK; 2 Department of Biology, Allergan Inc., Irvine, CA, USA; 3 Divisions of Cancer Studies, University of Birmingham Medical School, Edgbaston, Birmingham B15 2TT, UK; 4 Department of Urology, Queen Elizabeth Hospital, Birmingham B15 2TH, USA; 5 Retinoid Research, Department of Chemistry, Allergan Inc., Irvine, CA, USA

**Keywords:** RAR antagonists, retinoic acid receptors, prostate cancer, growth inhibition, apoptosis

## Abstract

Screening of synthetic retinoids for activity against prostate carcinoma cell lines has identified antagonists of retinoic acid receptors (RARs) as potent growth inhibitors (Hammond *et al*, 2001, *Br J Cancer* 85, 453–462). Here we report that 5 days of exposure to a high-affinity pan-RAR antagonist (AGN194310) abolished growth of prostate carcinoma cells from 14 out of 14 patients, with half-maximal inhibition between 200 and 800 nM. It had similar effects (at ∼250 nM) on the prostate carcinoma lines LNCaP, DU-145 and PC-3. AGN194310 inhibited the growth of normal prostate epithelium cells less potently, by 50% at ∼1 *μ*M. The growth of tumour cells was also inhibited more than that of normal cells when RAR*β* together with RAR*γ*, but not RAR*α* alone, were antagonised. Treatment of LNCaP cells with AGN194310 arrested them in G1 of cell cycle within 12 h, with an accompanying rise in the level of p21^waf1^. The cells underwent apoptosis within 3 days, as indicated by mitochondrial depolarisation, Annexin V binding and DNA fragmentation. Apoptosis was caspase-independent: caspases were neither cleaved nor activated, and DNA fragmentation was unaffected by the pan-caspase inhibitor Z-VAD-FMK. The ability of AGN 194310 to induce apoptosis of prostate cancer cells and its differential effect on malignant and normal prostate epithelial cells suggests that this compound may be useful in the treatment of prostate cancer.

Retinoids are important modulators of the survival, growth, and differentiation of normal and malignant cells. Screening of synthetic analogues has identified compounds that display significant potential as preventive and therapeutic anticancer agents (reviewed in Altucci and Gronemeyer, 2001; Fontana and Rishi, 2002; Hammond *et al*, 2002; Ortiz *et al*, 2002; Sun and Lotan, 2002). In particular, compounds have been identified that provoke growth arrest and/or apoptosis in cells from malignant and common carcinomas for which new treatments are needed. These include prostate cancer, the most common noncutaneous male cancer (reviewed in Hammond *et al*, 2002). After androgen ablation therapy, prostate cancer typically progresses to late stage androgen-independent disease that is incurable (Kirby, 1996; Ismail and Gomella, 1997; Landis *et al*, 1998).

Recent enthusiasm for treating various malignancies with retinoids has been fuelled by the characterisation of a variety of synthetic retinoids with diverse modes of action. These retinoids fall into two main classes, termed classical and novel (Fontana and Rishi, 2002). ‘Classical’ retinoids bind to and activate (or inactivate) the retinoic acid receptors (RAR*α*, *β* and *γ*) and retinoid X receptors (RXR*α*, *β* and *γ*). The ‘novel’ retinoids are synthetic retinoid-related molecules (RRMs) that exert at least some of their biological effects through RAR- and RXR-independent pathways (Fontana and Rishi, 2002; Ortiz *et al*, 2002). RRMs induce apoptosis in several types of malignant cells, including lung, cervical, breast and ovarian carcinomas and melanomas (Shao *et al*, 1995; Oridate *et al*, 1997; Piedrafita and Pfahl, 1997; Spanjaard *et al*, 1997; Sun *et al*, 1997; Li *et al*, 1998; Wu *et al*, 1998; Holmes *et al*, 2003).

ATRA and synthetic retinoid receptor agonists are not very effective in inducing growth arrest and/or apoptosis in prostate cancer cell lines such as LNCaP, DU-145 and PC-3: high concentrations (∼1–10 *μ*M) are generally needed (Gao *et al*, 1999; Hammond *et al*, 2001). When Lu *et al* (1999) screened more than 100 retinoids, three RAR*γ* agonists were the most active, but concentrations of 1–10 *μ*M were needed for complete growth inhibition. Similarly, we observed little effect of specific agonists of RAR*α* and RAR*βγ* on colony formation by the above cell lines (Hammond *et al*, 2001). In clinical studies, 13-*cis* retinoic acid (isotretinoin) (with or without interferon-*α*) is only modestly effective against prostate carcinoma (DiPaolo and Aisner, 1999; Kelly *et al*, 2000; Shalev *et al*, 2000).

By contrast, antagonists of RAR*αβγ* and RAR*βγ* cause striking cell growth arrest and inhibition of colony formation in LNCaP, PC-3 and DU-145 cells. For example, the pan-antagonist AGN194310 inhibits colony formation at around 50 nM – it is around 20-fold more potent than ATRA, and promotes growth arrest and apoptosis (Hammond *et al*, 2001). AGN194310 also inhibited the growth of primary prostate carcinoma cells from two patients more effectively than ATRA (Hammond *et al*, 2001). That RAR antagonists suppress the growth of epithelial carcinomas more effectively than receptor agonists is also suggested by a report that the weak RAR antagonist MX718 inhibits the growth of breast carcinoma lines more potently than ATRA (Fanjul *et al*, 1998). Retinoid receptor antagonists may therefore have a potential for use in the treatment of human prostate and breast cancers.

We therefore compared the effects of the pan-RAR antagonist AGN194310 on primary cell cultures established from 14 patients with prostatic carcinoma and on normal prostate epithelial cells and fibroblasts. Its more potent effect on patients' prostate carcinoma cells than normal prostatic epithelium indicates that this agent may be useful in prostate cancer therapy.

## MATERIALS AND METHODS

### Retinoids

The pan-RAR antagonist (AGN194310), an RAR*βγ* antagonist (AGN194431) and a specific RAR*α* antagonist (AGN196996), were synthesised at Allergan Inc. (Irvine, CA, USA). Their structures, receptor binding and transactivation properties have been described (Hammond *et al*, 2001). *K*_i_ values for binding of the antagonists to RAR*α*, *β* and *γ*, respectively, are AGN194310: 3, 2 and 5 nM; AGN194431: 300, 6 and 20 nM; and AGN196996: 2, 1087 and 8523 nM. These compounds show no activity in transactivation assays, but instead block the gene transcriptional activity induced by ATRA and other RAR agonists. To control for the detection of apoptosis and related events we used a RRM AGN193198 that induces apoptosis in a wide variety of cells through rapid caspase activation. AGN193198 does not bind to RARs and RXRs (Keedwell *et al*, 2004). Stock solutions were prepared at 10 mM in 50% ethanol/50% dimethysulphoxide and stored at −20°C.

### Cell lines and cell culture

Cultures of patients' carcinoma cells were established and maintained in serum-free medium and, for comparison, the cell lines investigated were grown serum-free or in low serum. Serum-free grown sublines of the prostate carcinoma cell lines LNCaP, PC-3 and DU-145 (serum stocks from ATCC, Rockville, MD, USA) have been described previously (Hammond *et al*, 2001). These lines were grown in RPMI 1640 medium (Gibco-BRL, Paisley, UK) containing antibiotics (100 U ml^−1^ penicillin and 100 *μ*g ml^−1^ streptomycin) and supplemented with insulin, transferrin, selenium dioxide, linoleic acid and bovine serum albumin (ITS^+^, Sigma, Poole, UK). The breast carcinoma lines MDA-MB-231 and MCF-7 (provided by Dr K Colston, St Georges Hospital, London) were adapted to growth in serum-free medium (MDA-MB-231) and in RPMI 1640 medium supplemented with 0.5% foetal bovine serum and antibiotics (Gibco-BRL, MCF-7) as described before (see Hammond *et al*, 2001). A subline of the promyeloid cell line HL60 has been grown long-term in serum-free (ITS^+^) medium (Bunce *et al*, 1995). Cells were grown at 37°C in a humidified atmosphere of 5% CO_2_ in air, and adherent cells were passaged by trypsinising with trypsin-EDTA (Gibco-BRL).

### Growth of primary cell cultures

Core biopsies were obtained from patients undergoing investigation for suspected prostatic carcinoma. Consent for the use of this material for research was obtained from the South Birmingham Local Research Ethics Committee of the Birmingham Health Authority. Informed consent was also obtained from the patients. Histological reports were obtained from the Department of Pathology, The Medical School, University of Birmingham, and confirmed that the biopsies contained adenocarcinoma. Slides and cases were also reviewed by one of the investigators (LAH).

Cultures were established as described by Peehl *et al* (1991), using the serum-free medium Prostate Epithelial Cell Growth Medium (PrEGM) supplemented with SingleQuots (BioWhittaker, Wokingham, UK). The biopsies were collected in ice-cold Hanks's buffered salt solution (HBSS) without phenol red (Gibco-BRL). Biopsies were washed 3 × with HBSS, and cut into very small (<1 mm^3^) pieces using a sterile scalpel blade. Cultures were established by placing the pieces of tissue into a 25 cm^2^ collagen 1-coated flask (Greiner, Stonehouse, UK) containing 1 ml of complete PrEGM medium. The time from collection to setting up the cultures was ∼3 h. By day 4, the pieces of tissue had attached to the flask, and 4 ml of PrEGM were added. Cells were subcultured at 80–95% confluence by using trypsin/EDTA, which was inactivated by adding a 10-fold greater volume of complete PrEGM. Cells were pelleted by centrifugation, and set in fresh collagen-1-coated flasks. Plates coated with collagen 1 (Greiner, Stonehouse, UK), and PrEGM were used for assays.

A primary culture of normal prostate epithelium cells (PrEC) was purchased from Cambrex Bio Science (Wokingham, UK). These cells test positive for cytokeratin (clone 8.13). The culture has a doubling time of 18–24 h, and undergoes ∼15 population doublings. Normal prostate fibroblasts grew out rapidly from a prostate biopsy that was nonmalignant, and the established culture consisted entirely of spindled fibroblast cells. The normal prostate epithelial cells and fibroblasts were grown as above.

### Analysis of the effects of antagonists on cell growth

Trypsinised cell suspensions were plated into a 96-well microtitre plate at 400 cells per well in 100 *μ*l of ITS^+^ medium (cell lines) or PrEGM (primary cells). Typically, these cells grow exponentially with doubling times between 20 and 24 h. Cells were treated, in triplicate, with retinoids immediately and at day 2 by replacing the medium. The number of viable cells was assessed at day 5 by measuring cellular ATP levels using the Vialight HS High Sensitivity Cell Proliferation/Cytotoxicity Kit according to the manufacturer's instructions (Lumi Tech, Nottingham, UK), using a Berthold LB953 luminometer. Vehicle alone had no effect on any of the cells tested.

Cell cycle status was measured by staining harvested cells with propidium iodide (PI, Molecular Probes, Eugene, OR, USA) in buffer (10 *μ*g ml^−1^ PI in 1% (w v^−1^) tri-sodium citrate, 0.1% (v v^−1^) Triton X100, 100 *μ*M NaCl). The distribution of cells between phases of the cell cycle was determined using a Becton-Dickinson Flow Cytometer and CellFIT Cell-Cycle Analysis software.

### Measurements of apoptotic events

Bulk cultures of LNCaP cells and primary prostate carcinoma cells were seeded at 5 × 10^5^ cells per 75 cm^2^ flask and AGN194310 was added immediately. Cells in suspension and adherent cells, harvested by trypsinisation, were pooled and apoptotic cells were identified by the TUNEL assay (Gorczyca *et al*, 1993). An FITC-conjugated antibody to bromodeoxyuridine (Becton-Dickinson & Co., Mountain View, CA, USA) was used to identify cells labelled with bromodeoxyurindine triphosphate and fluorescence was measured by FACS analysis at 510–550 nm.

Changes to the mitochondrial membrane potential after treatment of bulk cultures of cells with AGN194310 were measured by incubating harvested cells with 5 *μ*g ml^−1^ of the fluorescent probe JC-1 (5,5′,6,6′-tetrachloro-1,1′,3,3′-tetrabenzimidazolylcarbocyanine iodide, Eastman Kodak Co., Rochester, NY, USA) for 20 min at 37°C in an atmosphere of 5% CO_2_. After washing twice in phosphate-buffered saline for 10 min, cells were analysed on a Becton-Dickinson FACS (Salvioli *et al*, 1997).

Binding of Annexin V was determined as follows. LNCaP cells were plated onto 24-well plates (6000 cells per well) in medium containing 1 *μ*M AGN194310 or the equivalent amount of vehicle, and they were cultured for 1, 2 or 3 days. After careful removal of the medium, 200 *μ*l of buffer (1 × PBS, 5 mM CaCl, 140 mM NaCl) containing 3 *μ*g ml^−1^ Annexin V (Roche, Indianapolis, IN, USA) and 20 *μ*g ml^−1^ PI were added to each well and incubated in the dark for 15 min. Fluorescent microscopy was performed using an Axiovert 100 fluorescent microscope (Zeiss, Jena, Germany) with Image-Pro 4.5 software (Media Cybernetics, Silver Spring, MD, USA) for image capture and overlay.

For Fluorescence-Activated Cell Sorter (FACS) analysis of the levels of apoptosis, LNCaP cells were plated onto 60 mm dishes (1 × 10^5^ cells per dish) and treated with 0.1, 0.5, or 1 *μ*M AGN194310. Apoptosis was evaluated by Annexin-V-FITC/PI double staining using the Apoptosis Detection Kit (Oncogene, San Diego, CA, USA), according to the manufacturer's instructions. Flow cytometry was performed with a FACS Calibur (Becton Dickinson, Sunnyvale, USA), and Cell Quest Pro software. Single stain controls were performed for each analysis.

### Caspase studies

The pan-caspase inhibitor Z-VAD-FMK (R&D Systems, Abingdon, UK) was used to investigate the involvement of caspases in AGN194310-induced apoptosis. A 20 mM stock solution was prepared in dimethysulphoxide. Z-VAD-FMK was added at 50 *μ*M to bulk cultures of cells 1 h before adding AGN194310, and cells were monitored using the TUNEL assay.

Fluorometric assays for caspase activity were conducted in 96-well microtitre plates. In total, 50 *μ*l of assay buffer (20 mM HEPES, pH 7.5, 10% glycerol, 2 mM dithiothreitol) containing 50 *μ*M of peptide substrates for caspase-3 (DEVD-AFC), caspase-8 (IETD-AFC), or caspase-9 (LEHD-AFC) (all from Biovision Inc., Mountain View, CA, USA) were added to each well. In total, 50 *μ*l of cell lysate were added to initiate the reactions. Backgrounds were measured in wells that contained assay buffer, substrate and lysis buffer. Fluorescence was measured on a CytoFlour 4000 fluorescence plate reader (Applied Biosystems) set at 400 nm excitation and 508 nm emission. Caspase activities were calculated as fold increases relative to control wells.

Antibodies to caspase-3 (Stressgen), caspase-8 (Cell Signalling Technology) and caspase-9 (Cell Signalling Technology) were used to stain blots to detect cleaved (active) forms of these enzymes. Cells (1 × 10^7^ ml^−1^) were lysed in ice-cold buffer (5 mM EDTA, 150 mM NaCl, 1% Triton X-100, 100 *μ*M Na_3_VO_4_, 2 mM phenylmethylsulphonyl fluoride, 10 *μ*g ml^−1^ leupeptin, 50 mM Tris, pH 7.4) for 30 min with gentle rotation at 4°C. Lysates were clarified by centrifugation at 16 000 × *g* for 15 min at 4°C, resolved on SDS–PAGE, and transferred onto PVDF membranes for immunostaining. Immunoreactive proteins were visualised by enhanced chemiluminescence.

### Immunodetection of p21^waf1^ and p27^kip1^

Blots of cell lysates were stained with antibodies to p21^waf1^ (sc-397, Santa Cruz Biotechnology, Inc.) and p27^kip1^ (AHZ0462, Biosource), and immunoreactive proteins were visualised by enhanced chemiluminescence.

### Data manipulation and statistical analyses

At least three experiments of each type were performed with triplicate replicates. Results are expressed as means±standard errors of means (s.e.m.). The statistical significance between groups of data was analysed by the Student's *t*-test using the SigmaStat™ (version 8.0) statistical software package. Dose–response curves for compounds were plotted using the kinetics module of the Sigmaplot™ (version 8.0) graphics software package.

## RESULTS

### The pan-RAR antagonist AGN194310 is a potent growth inhibitor of prostate carcinoma cells

**Figure 1:**
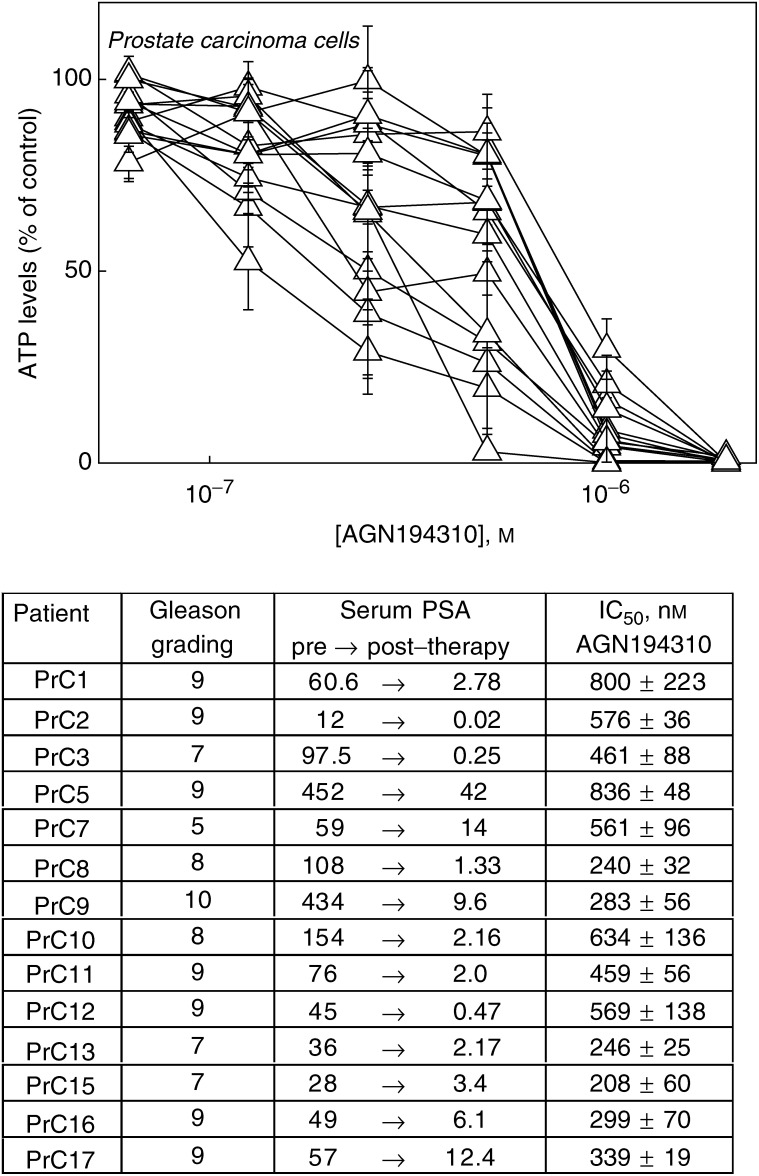
AGN194310 potently inhibits the growth of patients' prostate carcinoma cells. Activity of AGN194310 against carcinoma cells from core biopsies of 14 patients with prostatic carcinoma was measured by seeding cells into wells of a microtitre plate, treating with agent immediately and at day 2, and measuring cellular ATP levels at day 5. The top panel shows the titration curves obtained for each of the patients' cells. The IC_50_ values shown in the table are means±s.e. of data obtained from three separate experiments.

Core biopsies of prostate tissue were obtained from patients attending a diagnostic urological clinic and in whom there was a high clinical suspicion of prostatic carcinoma. Following histological examination of the cores and reporting of invasive carcinoma, the patients received androgen ablation (cyprotone acetate and zolodex). All of the patients responded to therapy as revealed by substantial reductions in their serum levels of prostate-specific antigen (PSA, see Figure 1

).

Epithelial carcinoma cells were grown from the above core biopsies of 14 patients with prostatic carcinoma. The biopsies contained extensive carcinoma, and cells in the cultures established were almost entirely polygonal, epithelial cells. The cells expressed prostate-specific antigen, as revealed by immunocytochemical staining. Their epithelial nature was confirmed by positive immunocytochemical staining for cytokeratin. Cultures contained few fibroblasts and stromal (smooth muscle) cells, as revealed by immunostaining for cytokeratin and smooth muscle actin.

Cells from each of 14 patients were tested, at passage 2, for sensitivity to AGN194310 and ATRA. In the microtitre plate assay, AGN194310 completely inhibited growth at 1–2 *μ*M. There was some variability in the sensitivity of individual patients' cells to AGN194310, with concentrations that reduced cell numbers by 50% (IC_50_ values) ranging from ∼200 to ∼800 nM (Figure 1). IC_50_ values were not related to Gleason grading or to pre- and post-therapy PSA levels (see Figure 1). ATRA (up to 2 *μ*M) had little effect on cells from 13 patients, and an IC_50_ value of 1 *μ*M was obtained for cells from patient PrC2 (data not shown).

### AGN194310 inhibits the growth of human prostate cancer cells more potently than normal prostate epithelium

We compared the activities of AGN194310 against three prostate carcinoma lines, a patient's primary carcinoma cells and normal prostate-derived epithelial cells and fibroblasts. Prostatic fibroblasts were grown from a nonmalignant biopsy: the culture contained no epithelial cells.

Figure 2A

**Figure 2:**
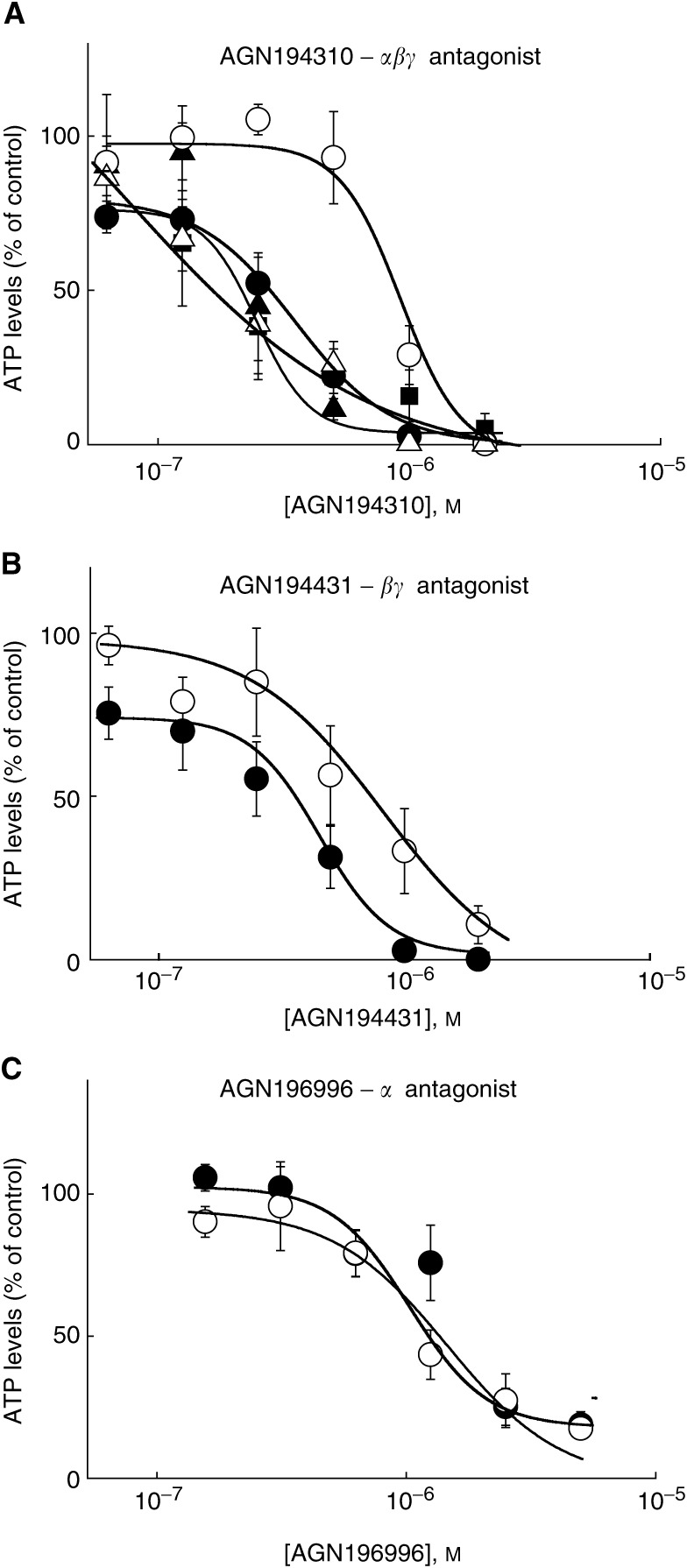
Pan-RAR antagonist AGN194310 affects the growth of prostate carcinoma cells more than that of normal prostate epithelium. The top panel shows the activity of the pan-RAR antagonist AGN194310 against LNCaP cells (closed circles), PC-3 cells (closed triangles), DU-145 (squares) a patient's primary carcinoma cells (open triangles) and normal prostate epithelium (open circles). The lower panels show the activities of antagonists of RAR*βγ* (AGN194431) and RAR*α* (AGN196996) against LNCaP cells (closed circles) and normal prostate epithelium (open circles). Activities were measured by seeding cells into wells of a microtitre plate, treating with agents immediately and at day 2, and measuring cellular ATP levels at day 5. Data are means±s.e. of values obtained from six (**A**), four (**B**) and three (**C**) experiments. The *P*-values obtained when the IC_50_ value for AGN194310 against normal prostate epithelium was compared with IC_50_ values against LNCaP, PC-3 and DU-145 cells are 0.008, 0.001 and 0.009, respectively. Comparison of the IC_50_ values for AGN194431 against normal prostate epithelium and LNCaP cells gave a *P*-value of 0.1, and a *P*-value of 0.4 was obtained for this comparison for AGN196996.

shows the data from parallel studies of AGN194310 activity against LNCaP, PC-3 and DU-145 cells, against epithelial primary cultures from a patient with prostate carcinoma, and against normal prostate epithelium cells. AGN194310 potently reduced cell numbers when the three prostate carcinoma cell lines were grown in microtitre wells, with IC_50_ values of 343±78 nM for LNCaP cells, 183±76 nM for PC-3 cells and 243±56 nM for DU-145 cells. In total, 500 nM AGN194310 reduced cell numbers by ∼80% in the cultures of the three prostate carcinoma lines and the patient's primary cells, and it had no effect on the normal prostate epithelium cells. The prostate carcinoma cell lines were approximately four times as sensitive to AGN194310 as normal prostate epithelial cells (*P*=0.004), and comparison of the IC_50_ value for normal cells *vs* individual values for LNCaP, PC-3 and DU-145 cells gave *P*-values of 0.008, 0.001 and 0.009, respectively. Moreover, the dose–response curve for the normal prostate epithelium cells was displaced to the right of the dose–response curves for the carcinoma cells from all of the 14 patients' carcinoma cells (compare Figures 1 and 2). The normal prostate fibroblasts (IC_50_ ∼0.8 *μ*M), like the normal prostate epithelial cells (IC_50_ ∼1 *μ*M, *P*=0.4 for comparison of these two IC_50_ values), were less sensitive to AGN194310 than the prostate carcinoma cells.

To compare the involvement of the three RARs in controlling the growth and survival of malignant and normal cells, antagonists of RAR*βγ* (AGN194431) and of RAR*α* (AGN196996) were also titrated against LNCaP and normal prostate epithelial cells (Figure 2B and C). As reported previously (Hammond *et al*, 2001), the RAR*βγ* antagonist was as potent as the pan-antagonist against LNCaP cells (IC_50_=291±76 nM), and the RAR*α* antagonist (IC_50_=1.8±0.3 *μ*M) was less potent (compare Figures 2A, B and C). The RAR*βγ* antagonist was more active against LNCaP cells than against the normal prostate epithelium cells (*P*=0.1 for comparison of IC_50_ values), but this differential effect was less than for the pan-specific antagonist AGN194310. The RAR*α* antagonist did not discriminate between LNCaP cells and normal prostate epithelial cells (*P*=0.4 for comparison of IC_50_ values).

### AGN194310 provokes growth arrest in G1

**Figure 3:**
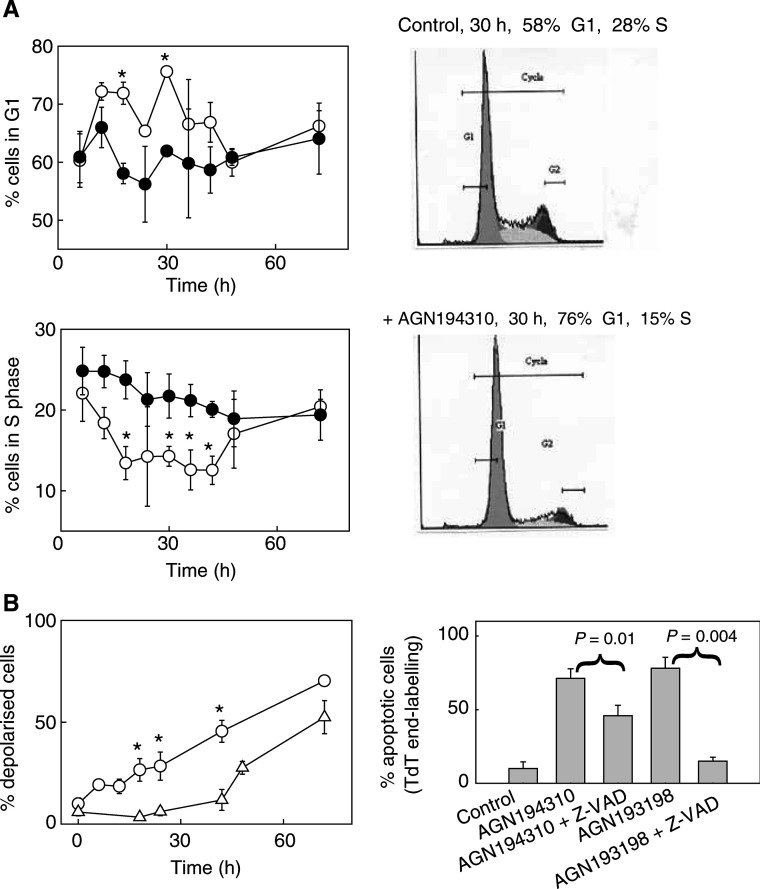
AGN194310 causes LNCaP cells to arrest in G1 and later undergo apoptosis. (**A**) Kinetics of changes to the cell cycle status of LNCaP cells treated with 1 *μ*M AGN194310 (open circles) as compared to the status of control (untreated) cultures (closed circles). Cell cycle status was measured after staining cells with propidium iodide, and representative profiles are shown. (**B**) Time course for the induction of mitochondrial membrane depolarisation (open circles) and the appearance of DNA-strand breaks (TUNEL assay, open triangles) when LNCaP cells were treated with 1 *μ*M AGN194310. The right panel shows that AGN194310-induction of DNA-strand breaks was largely unaffected by treating LNCaP cells with the pan-caspase inhibitor (at 50 *μ*M) for 1 h before adding 1 *μ*M AGN194310 for 3 days. By contrast, Z-VAD-FMK blocked AGN193198-induced apoptosis. Data are means±s.e. of values obtained from three time course experiments, and five experiments using the pan-caspase inhibitor. ^*^Denotes *P*-values <0.05.

Previously we reported that AGN194310 induces growth arrest and apoptosis of LNCaP cells (Hammond *et al*, 2001). Here we report detailed kinetics of these processes. Treatment of LNCaP cells with 1 *μ*M AGN194310 first led to a substantial increase in the proportion of cells in G1 of cell cycle, which reached a maximum level that was maintained from ∼12 h (see Figure 3A

), with a corresponding decline in the population of S phase cells.

**Figure 4:**
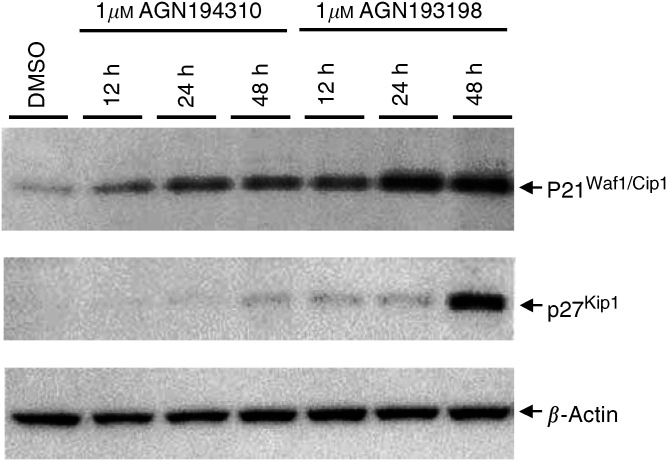
AGN194310 increases p21^waf1^ protein levels in LNCaP cells. Blots of cell extracts were immunostained with antibodies to p21^waf1^ and p27^kip1^. The figure shows that p21, but not p27, increased in level when LNCaP cells were treated with AGN194310. To control for the detection of p27, LNCaP cells were treated with the RRM AGN193198.

P21^waf1^ was detected at low levels in LNCaP cells, and AGN194310 (1 *μ*M) provoked an increased expression from ∼12 h onwards (Figure 4

). The accumulation of p21^waf1^ occurred at about the same time as cells accumulated in G1 of cell cycle. P27^kip1^ was not detectable in untreated LNCaP cells, and was present at a very low level even after treating for 48 h with AGN194310. The detectability of induced expression of p27^kip1^ was controlled by observing a large rise (at 48 h) upon treatment of LNCaP cells with AGN193198. This compound also induced a very rapid and substantial rise in the level of expression of p21^waf1^.

### Apoptosis follows G1 arrest

**Figure 5:**
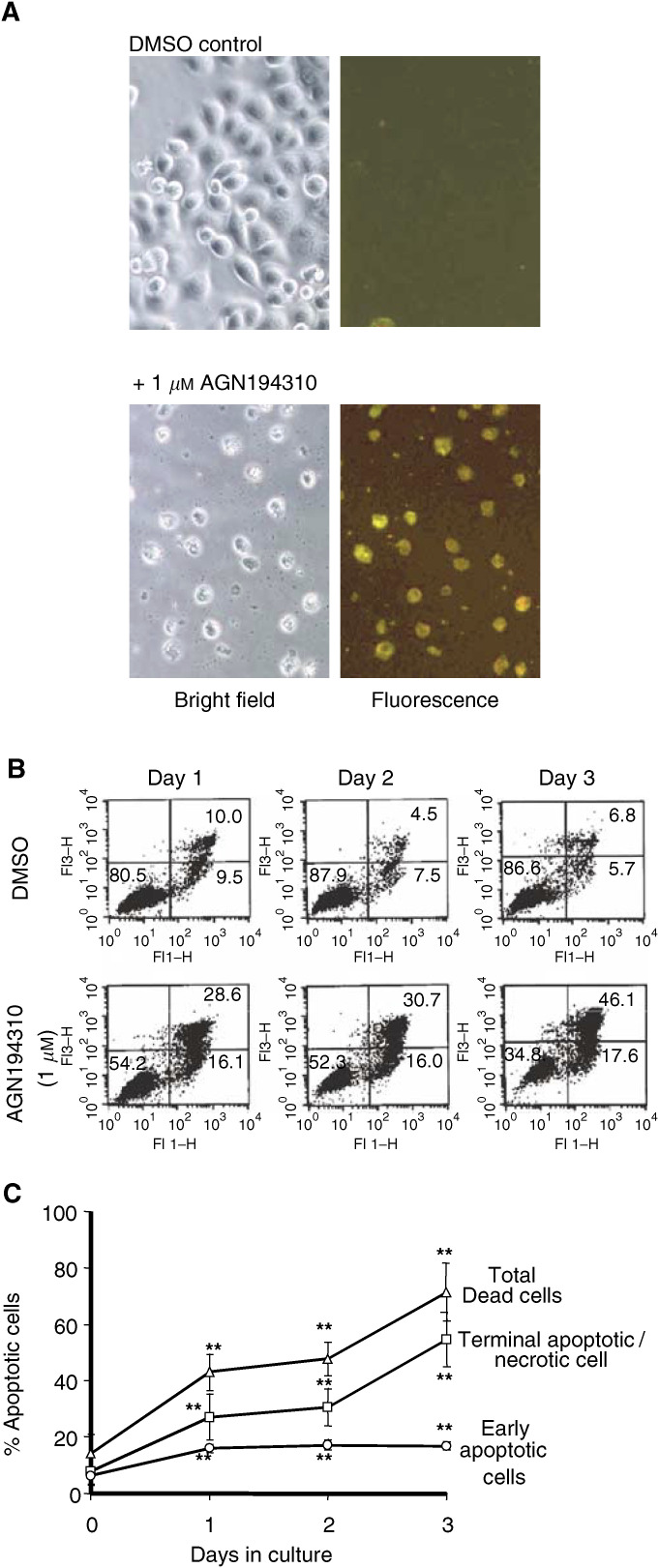
LNCaP cells undergoing AGN194310-induced apoptosis bind Annexin V. LNCaP cells were treated with 1 *μ*M AGN194310, or vehicle control (DMSO) and stained with Annexin V and propidium iodide. Fluorescence microscopy at day 3 (**A**) reveals early apoptotic cells (Annexin V +ve; green), as well as late apoptotic/necrotic cells (Annexin V +ve/propidium iodide +ve; yellow-red) in AGN194310-treated cultures. (**B**) Representative FACS cytograms of stained cells. Viable cells (Annexin V and propidium iodide −ve) are in the lower left-hand quadrant. Early apoptotic cells (Annexin V +ve/propidium iodide −ve) are in the lower right-hand quadrant. Terminal apoptotic/necrotic cells (Annexin V +ve/propidium iodide +ve) are in the upper right-hand quadrant. (**C**) Results of the flow cytometry analyses are summarised. Data are means±s.e. of values from at least three experiments. ^**^Denotes *P*-values <0.001.

Levels of apoptosis were monitored by following changes in the mitochondrial potential, by the appearance of phosphatidylserine at the cell surface, and by detection of DNA-strand breaks (Figures 3B and 5

). Apoptosis was induced progressively in AGN194310-treated cells – 50% of the cells had depolarised mitochondria by 40–50 h, and cells displaying DNA-strand breaks accumulated progressively after ∼40 h. After 3 days, ∼70% of cells showed mitochondrial depolarisation, DNA strand-breaks were detected in ∼50% of cells, and 60% of the cells had exposed phosphatidylserine at their cell surface and so bound Annexin V (Figure 5).

### AGN194310-induced apoptosis in LNCaP cells is caspase-independent

To investigate the role of caspases in AGN194310-induced apoptosis, LNCaP cells were treated for 1 h with the pan-caspase inhibitor Z-VAD-FMK (50 *μ*M) prior to adding 1 *μ*M AGN194310. The inhibitor only slightly reduced the proportion of cells showing DNA strand-breaks at day 3 (Figure 3, *P*-value 0.01). The Z-VAD-FMK used was biologically active. The RRM AGN193198 potently induces apoptosis in a variety of cells, and ∼80% of treated LNCaP cells showed mitochondrial depolarisation and DNA fragmentation by day 3. Z-VAD-FMK (50 *μ*M) largely prevented this apoptosis (15% apoptotic cells, *P*-value 0.004, Figure 3B).

**Figure 6:**
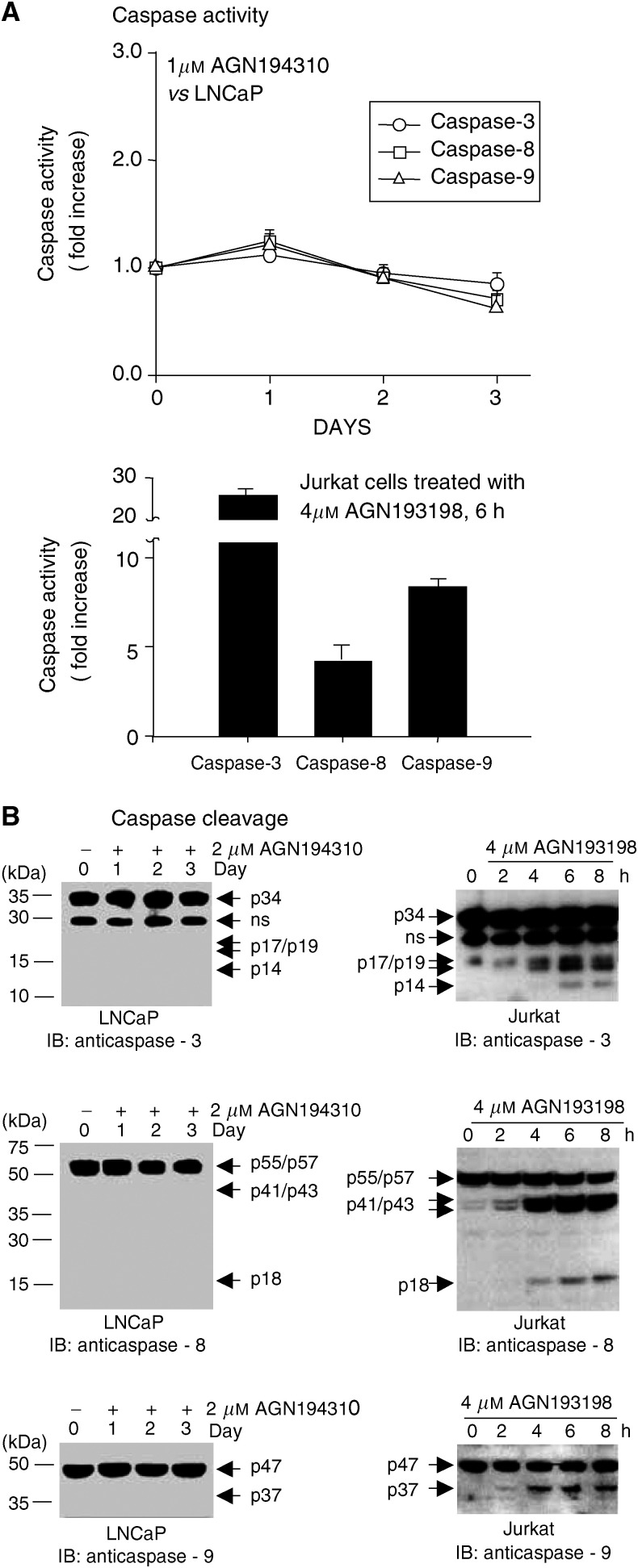
Caspases are neither cleaved nor activated in AGN194310-treated LNCaP cells. (**A**) that caspases-3, -8 and -9 were not activated in LNCaP cells treated with 1 *μ*M AGN194310. To control for detection of activated enzymes, Jurkat T cells were treated for 6 h with 4 *μ*M AGN193198 (bottom panel). Enzyme activities were measured using peptide substrates. (**B**) Caspases-3, -8 and -9 were not cleaved in AGN194310 (1 *μ*M)-treated LNCaP cells, as revealed by immunostaining blots of cell extracts. To control for detection of cleaved (active) forms of caspases, Jurkat T cells were treated with 4 *μ*M AGN193198 (right immunoblots).

Caspases-3, -8 and -9 were not cleaved or activated when LNCaP cells were treated with 1 *μ*M AGN194310 (Figure 6

). Enzyme activities remained unchanged, and immunostaining of cell extracts did not detect cleaved forms. In parallel control experiments, 6 h of treatment of Jurkat T cells with 4 *μ*M AGN193198 induced 25-, four- and eight-fold increases in the activities of caspases-3, -8 and -9 (Figure 6A, lower panel), and cleaved forms of these enzymes were readily detectable by 4 h (Figure 6B, right panel).

### HL60 leukaemia and breast carcinoma cells are less sensitive to AGN194310 than prostate cancer cells

Table 1

**Table 1:** Growth inhibitory activity of the pan-RAR antagonist AGN194310 against various cell types

**Cells**	**IC_50_ of AGN194310 (nM)**
*Prostate carcinoma cells*	
Patients' primary cells	208±60 to 836±48
LNCaP	343±78
PC-3	183±76
DU-145	243±56
	
*Other malignant cells*	
MCF-7 (breast carcinoma)	>2000
MDA-MB-213 (breast carcinoma)	781±27
HL60 (promyeloid leukaemia)	1338±318
	
*Normal prostate cells*	
Epithelium	960±134
Fibroblasts	786±12

IC_50_ values were determined using the microtitre plate assay, and are means±s.e. of values obtained from at least three separate experiments.

compares the IC_50_ values obtained from titrations of AGN194310 against prostate carcinoma cells and other cell types that we tested. AGN194310 does not inhibit colony formation by single HL60 cells plated in microtitre plate wells (Hammond *et al*, 2001). When AGN194310 was screened for activity against HL60 cells plated in microtitre wells, an IC_50_ value of 1.3 *μ*M was obtained (data not shown). The breast carcinoma lines MCF-7 and MDA-MB-231 were less sensitive than the prostate carcinoma lines to growth inhibition by AGN194310. A 50% inhibition of growth was not achieved for MCF-7 cells at 2 *μ*M AGN194310, and an IC_50_ value of ∼800 nM was obtained for MDA-MB-231 cells.

## DISCUSSION

Finding new treatments for malignant carcinomas requires the identification of new agents that potently induce growth arrest and/or apoptosis, and that are more effective against malignant cells than normal cells. For example, the synthetic retinoid CD437 induces rapid apoptosis in human lung cancer cell lines but not in two types of normal lung epithelial cells (Sun *et al*, 2002), and it also induces apoptosis in malignant human epidermal keratinocytes but not in normal keratinocytes (Hail and Lotan, 2001).

Here, we have shown that the pan-specific RAR antagonist AGN194310 potently inhibits the growth of prostate carcinoma cells, which display an accumulation of p21^waf1^, are arrested in G1 of cell cycle, and then undergo apoptosis. Similarly, the RAR antagonist BMS453 causes accumulation of p21^waf1^ and G1 arrest of normal breast cells (Yang *et al*, 2001). The antiproliferative effects of several agents against prostate carcinoma cell lines have been attributed to upregulation of p21^waf1^ expression: for example, G1 accumulation of LNCaP in response to 1*α*, 25-dihydroxyvitamin D_3_ (Moffat *et al*, 2001; Yang *et al*, 2002). Prostate carcinoma cell lines that do not undergo growth arrest in response 1*α*, 25-dihydroxyvitamin D_3_ fail to upregulate p21^waf1^ (Moffat *et al*, 2001). Other agents that induce G1 arrest of prostate carcinoma lines, such as 12-*O*-tetra-decanoylphorbol-13-acetate, indole-3-carbinol and inositol hexaphosphate, also upregulate p21^waf1^ and p27^Kip1^, leading to decreases in cyclin-dependent kinase activities (Chinni *et al*, 2001; Sugibayashi *et al*, 2002; Singh *et al*, 2003).

The pan-specific RAR antagonist AGN194310 largely prevented the growth in microtitre wells of malignant prostate epithelium cells from androgen-responsive patients and of LNCaP, PC-3 and DU-145 cells at submicromolar concentrations. Previously, we have reported that this compound reduces viable cell numbers in flask cultures of LNCaP cells and of patients' prostatic carcinoma cells (Hammond *et al*, 2001). Also, AGN194310 potently inhibits colony formation by LNCaP (IC_50_ 16 nM), PC-3 (IC_50_ 18 nM) and DU-145 (IC_50_ 34 nM) cells (Hammond *et al*, 2001). That more AGN194310 is required to reduce cell numbers in microtitre wells is commensurate with the view that the colony formation assay is the more sensitive. Normal prostate epithelial cells were substantially less sensitive to AGN194310 than carcinoma cells. This compound may, therefore, have potential for killing tumour cells *in vivo* while sparing normal cells. Relative insensitivity of normal prostate fibroblasts, haemopoietic cells such as HL60, and breast carcinoma cells to AGN194310 (Hammond *et al*, 2001) also indicate that other cell types might be spared *in vivo*.

Several observations suggest that AGN194310 induces G1 arrest of prostate carcinoma cells through a mechanism that involves antagonism of multiple RARs, rather than some unrelated target molecule(s). The pan-specific RAR antagonist AGN194310 and the RAR*βγ* antagonist AGN194431 have different structures, but both inhibit colony formation by and growth in liquid culture of prostate carcinoma cells at low concentrations. It seems unlikely that these two compounds could both bind with similar high affinities to a second target. Moreover, the pan-RAR agonist TTNPB ((E)-4-[2-(5,6,7,8-tetrahydro-5,5,8,8-tetramethyl-2-naphthalenyl-1-propenyl]-benzoic acid) overcomes the growth inhibitory effect of AGN194310 (Hammond *et al*, 2001). It seems likely that AGN194310 abolishes RAR-mediated effects of agonistic retinoids that facilitate the proliferation of prostate carcinoma cells. These RAR-mediated effects must be achieved by a very low level of retinoid signalling that is caused by trace amounts of retinoids that the cells encounter while grown serum-free – very small amounts of retinoids may contaminate the medium constituents. Alternatively, the growth inhibitory effects of AGN 194310 may result from direct pharmacological effects involving assembly of RAR complexes containing corepresser molecules.

Previously, we argued that RAR*γ* antagonism may be sufficient to compromise the growth of LNCaP, PC-3 and DU-145 cells that are grown long-term serum-free, since these cells express only RAR*α* and RAR*γ* (Campbell *et al*, 1998; Hammond *et al*, 2001; and data not shown), and RAR*βγ* antagonism is as effective as pan-antagonism. The importance of RAR*γ* in maintaining growth and/or survival of prostate epithelium is supported by findings from RAR*γ*-null mice, the prostates of which show atrophy and squamous metaplasia (Lohnes *et al*, 1993). The RAR*α*-null mouse does not show these defects (Lufkin *et al*, 1993). However, we cannot exclude the biological involvement of a very low level of RAR*β* expression in the human cell lines and tumours. RAR*β* was not detected by immunoblotting cell extracts, but a band is just visible on immunoblots of extracts of serum-grown LNCaP cells that are known to express RAR*β* (Campbell *et al*, 1998; Hammond *et al*, 2001). Many studies have suggested a role for RAR*β* in modulating the growth and survival of prostate cancer cells (reviewed in Zhang, 2002). For example, stable expression of RAR*β* in RAR*β*-negative PC-3 cells increases their sensitivity to growth inhibition by agonistic retinoids (Campbell *et al*, 1998). In the present study, the difference in sensitivity of the malignant and normal cells to the RAR*βγ* antagonist was less than when RAR signalling was extinguished by the RAR*αβγ* antagonist. That gene expression regulated by RAR*γ* or RAR*β* might be more necessary to the growth of prostate carcinoma cells than their normal counterparts remains an interesting possibility.

Paradoxically, agonists and antagonists of RARs both provoke growth arrest of prostate carcinoma cells (Lu *et al*, 1999; Hammond *et al*, 2001). But, assuming that serum free-grown LNCaP, PC-3 and DU-145 cells express *α* and *γ*, we do not know how the various gene regulatory activities of these two receptors contribute to promoting and/or arresting the growth of cells. ATRA provokes G1 arrest and differentiation of HL60 cells (Brietman *et al*, 1980) via RAR*α*: this is the major receptor expressed in undifferentiated myeloid cells (Zhu *et al*, 2001), and resistance of myeloid cells to ATRA is related to dominant-negative RAR*α* mutations (Ding *et al*, 1998; Duprez *et al*, 2000). One possible explanation of the growth inhibitory activities of both agonists and antagonists against prostate carcinoma cells is that agonism of RAR*α* drives gene expression leading to cell growth arrest, but also that antagonism of RAR*γ* switches off expression of a molecule(s) that plays a role in facilitating cell proliferation. The relative contributions of positive and negative effects on cell growth may determine the final outcome.

Prostate carcinoma cells that growth-arrested in response to AGN194310 quickly underwent apoptosis. Most studies of apoptosis focus on caspases as critical elements: the steps leading to DNA fragmentation are generally considered to be caspase-3 initiated (Enari *et al*, 1999). Caspases have also been proposed as the major effectors of the action of RRMs (You *et al*, 2001; Lopez-Hernandez *et al*, 2003). However, the pan-caspase inhibitor Z-VAD-FMK did not substantially affect the induction of apoptosis by AGN194310, and caspases-3, -8 and -9 were neither cleaved nor activated. Hence, we conclude that AGN194310-provoked apoptosis is caspase-independent.

There is other evidence that caspases are not essential for apoptosis, nor is caspase activity sufficient to execute cell death (Lukovic *et al*, 2003). Endonuclease G is an apoptotic DNAase that is released from the mitochondria (Li *et al*, 2001), and etoposide-induced apoptosis in the HeLa cells involves caspase-independent activation of endonucleases (Torriglia *et al*, 1999). Indeed, triggering of caspase-independent cell death has been proposed as a means of combating cancer (reviewed in Sun *et al*, 2000; Mathiasen and Jaattela, 2002; Lewis *et al*, 2003).

Our studies suggest that AGN194310 may be useful in the treatment of prostate cancer, and this compound is under clinical development.
